# Update on human herpesvirus 7 pathogenesis and clinical aspects as a roadmap for future research

**DOI:** 10.1128/jvi.00437-24

**Published:** 2024-05-08

**Authors:** Rianne Verbeek, Linos Vandekerckhove, Jolien Van Cleemput

**Affiliations:** 1 HIV Cure Research Center, Faculty of Medicine and Health Sciences, Ghent University, Ghent, Belgium; Indiana University Bloomington, Bloomington, Indiana, USA

**Keywords:** HHV-7, viral pathogenesis, knowledge gaps, human herpesviruses

## Abstract

Human herpesvirus 7 (HHV-7) is a common virus that is associated with various human diseases including febrile syndromes, dermatological lesions, neurological defects, and transplant complications. Still, HHV-7 remains one of the least studied members of all human betaherpesviruses. In addition, HHV-7-related research is mostly confined to case reports, while *in vitro* or *in vivo* studies unraveling basic virology, transmission mechanisms, and viral pathogenesis are sparse. Here, we discuss HHV-7-related literature linking clinical syndromes to the viral life cycle, epidemiology, and viral immunopathogenesis. Based on our review, we propose a hypothetical model of HHV-7 pathogenesis inside its host. Furthermore, we identify important knowledge gaps and recommendations for future research to better understand HHV-7 diseases and improve therapeutic interventions.

## INTRODUCTION

**Fig 1:**
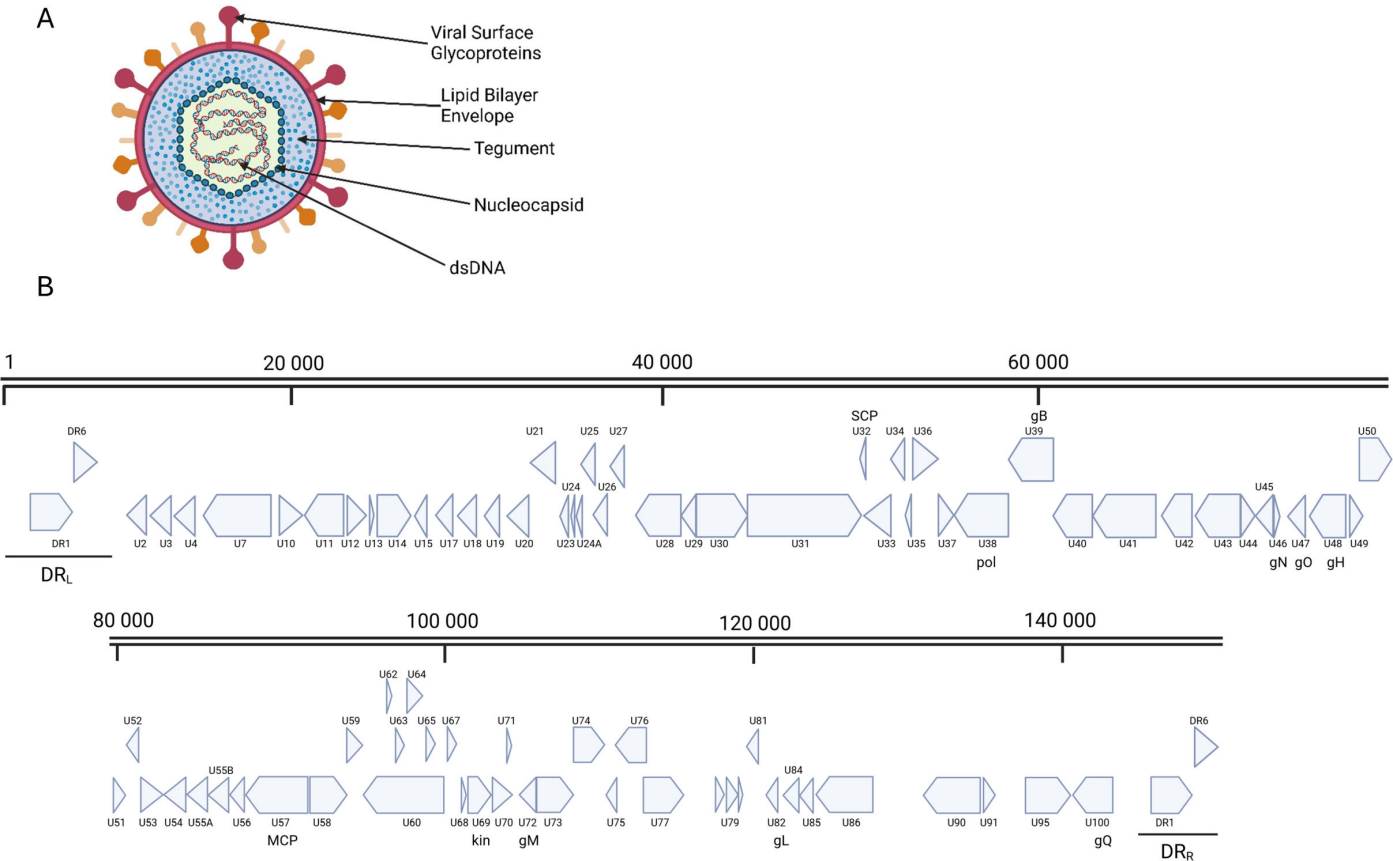
Human herpesvirus 7 (HHV-7) particle structure and genome. (**A**) Schematic overview of the HHV-7 particle structure with indication of major viral components. (**B**) Schematic representation of the genome arrangement of HHV-7 based on the NCBI reference genome NC_001716.2. DRL (left), DRR (right): direct repeats. SCP: small capsid protein, pol: DNA polymerase, gB: glycoprotein B, gN: glycoprotein N, gO: glycoprotein O, gH: glycoprotein H, MCP: major capsid protein, kin: serine/threonine protein kinase, gM: glycoprotein M, gL: glycoprotein L, gQ: glycoprotein Q. Scale bars represent the number of base pairs. Figure made using BioRender.com.

Human herpesvirus 7 (HHV-7) is a ubiquitous CD4^+^ T-lymphotropic virus that was first isolated from peripheral blood lymphocytes of a healthy individual in 1990 (1). As a member of the *Herpesviridae* family, *Betaherpesvirinae* subfamily, the DNA virus HHV-7 closely resembles human cytomegalovirus (HCMV or HHV-5) and even more so human herpesviruses 6A and 6B (HHV-6A and HHV-6B), here collectively referred to as “HHV-6” unless otherwise specified, with whom it shares the genus *Roseolovirus*. Along with the latter, primary HHV-7 infection is associated with childhood febrile syndromes, whether or not accompanied by a rash, classified as “the sixth disease” (2). Over 95% of human adults are HHV-7 seropositive due to prior infection and thus persistently infected with HHV-7 (3). Indeed, primary herpesvirus infection typically results in a persistent infection during which periods of latency are interspersed with periods of reactivation (4). Although HHV-7 infection is generally considered to be benign, an increasing number of studies link the virus to more severe clinical syndromes such as transplant complications and neurological defects. Still, the virus is one of the least studied human herpesviruses. Indeed, on March 6th, 2024, merely 904 full-text articles were found using the search term “HHV-7” in PubMed (https://pubmed.ncbi.nlm.nih.gov/), compared to 3,932 items for “HHV-6” and 46,033 for “HHV-5.” The viral genome and particle structure (Fig. 1), including the major differences with those of HHV-6, and specific HHV-7-related clinical syndromes have been reviewed before (2, 5–8). However, a recent comprehensive overview of the viral pathogenesis and associated clinical manifestations is lacking. Here, we summarize the current state of knowledge on HHV-7 infection in humans to outline a hypothetical model for the viral pathogenesis and highlight areas for future research.

## VIRAL LIFE CYCLE

**Fig 2:**
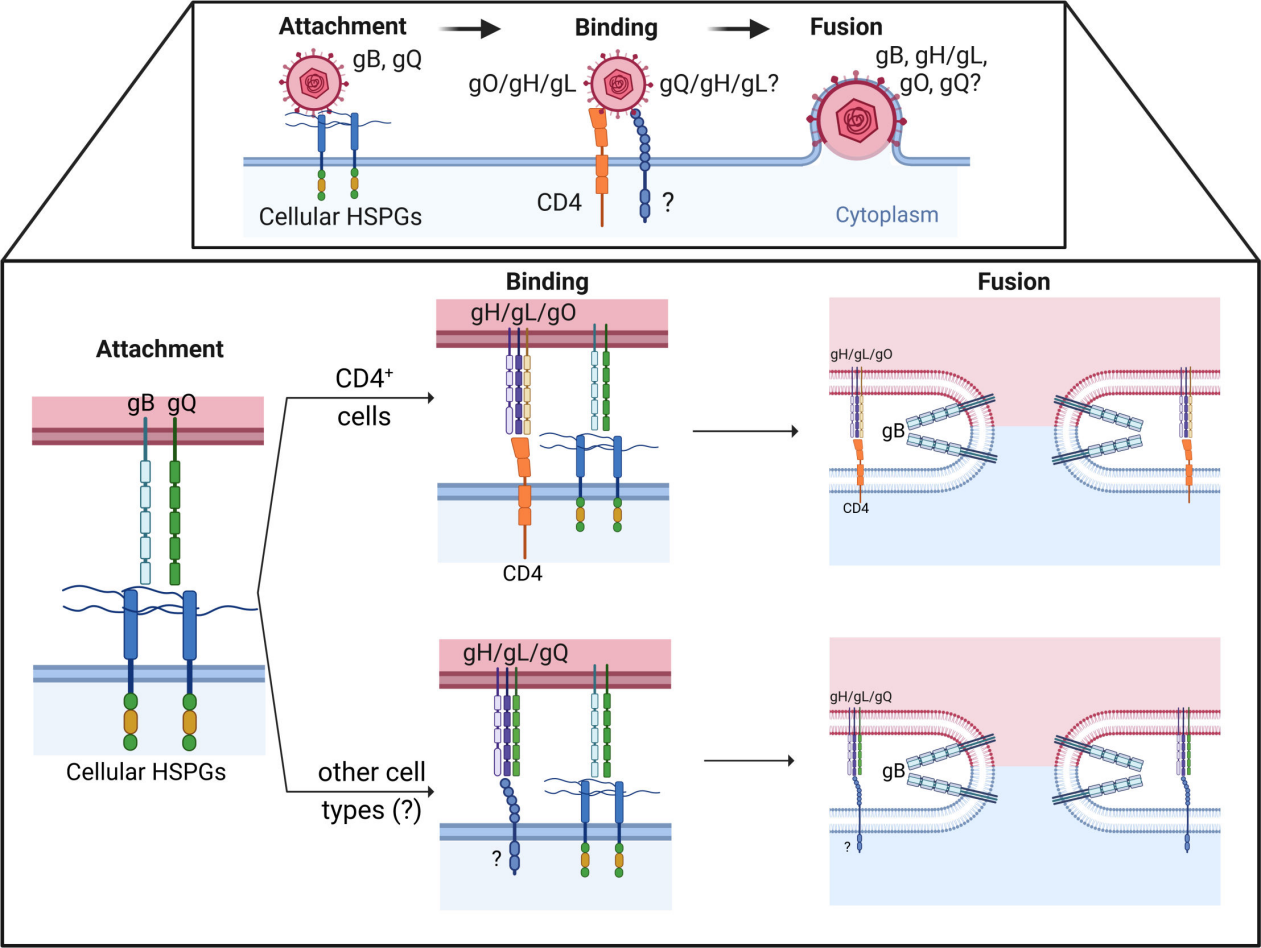
Hypothetical model of HHV-7 entry in host cells. Virus attachment to, binding to, and entry in host cells occurs through the engagement of viral ligands and host cell surface receptors (upper panel). Close-up of these different steps according to models proposed for CD4^+^ T cells and other cell types (lower panel). The figure was created using BioRender.com.

Herpesviral entry in host cells is mediated by interactions between viral envelope glycoproteins and molecules on the cell membrane. This complex process is divided into the following three steps: (i) virion attachment to the cell surface, (ii) virion interaction with a specific entry receptor, and (iii) virion internalization and membrane fusion. The studies of Black et al. (9) and Ablashi et al. (10) show transmission electron micrographs of these different steps during HHV-7 infection in lymphocytes. As illustrated in Fig. 2, HHV-7 initial adsorption to cells is likely mediated by the binding of viral envelope glycoproteins B and Q (gB and gQ) to cell-surface heparan sulfate proteoglycans (11, 12). Homologs of gB are found in all herpesviruses studied to date, but gQ is unique to HHV-6 and -7. The 65 kDa HHV-7 gQ is translated from multiply spliced mRNA encoded by ORF U100 (12, 13). In HHV-6, two transcripts of the U100 gene are produced, gQ1 (80 kDa) and gQ2 (37 kDa) (14). Whether this is also true for HHV-7 ORF100 gene products is unknown. Following initial attachment, HHV-7 virions firmly anchor onto a cellular receptor subsequently triggering fusion of the viral envelope and cellular membrane. CD4 is the sole known receptor for HHV-7. Indeed, overexpression of CD4 permits HHV-7 entry in non-permissive cell lines, while blocking CD4 using monoclonal antibodies or HIV gp120 inhibits HHV-7 entry (15–17). Still, additional unidentified cellular receptors likely also mediate HHV-7 entry, as the virus can productively infect cells lacking CD4 expression such as epithelial cells, endothelial cells, natural killer (NK) cells, megakaryocytes, dendritic cells, neurons, astrocytes, and oligodendrocytes (15, 16, 18–25). Notably, HHV-7 binding and entry are independent of HIV co-receptors CXCR4 and CCR5 (26, 27). Moreover, a low or mere expression of CD4 is not sufficient for productive viral infection, as CD4^+^ HeLa, Jurkat, and THP1 cells do not support productive viral replication (15, 28). Whether these cells are not susceptible and do not support viral entry or are not permissive due to a block in viral replication is unknown. The putative viral ligand for CD4 is still unidentified, but plausible candidates are viral envelope glycoproteins gH, gL, or gO (11, 17). Since fusion products between the extracellular domain of HHV-7 gB and the Fc domain of human immunoglobulin G heavy chain γ1 do not bind CD4^+^ T cells, gB likely does not engage CD4 (11). Co-expression of gB, gH, gL, and gO in HEK293T cells was necessary to induce membrane fusion and CD4 played a major role in this process, indicating that all four glycoproteins cooperate in the viral entry step (17). In general, herpesvirus gH and gL form a heterodimer complex that interacts with specific cell receptors which is then thought to induce a conformational change of the fusogen gB (pre- to post-fusion) to complete membrane fusion. In other betaherpesviruses (HCMV and HHV-6), gH/gL combines with additional viral envelope glycoproteins to form tri-, tetra-, and even pentamers to promote viral entry and provide receptor specificity (Table 1) (29, 30). Thus, we could speculate that HHV-7 may interact with CD4 through the engagement of the gH/gL/gO complex, subsequently triggering membrane fusion with the help of gB (11, 17, 31, 32). Alternatively, gH/gL/gQ and gB binding to putative receptors might also trigger viral entry into host cells, but evidence is currently lacking. In comparison, HHV-6 employs the multiprotein complex gH/gL/gQ1/gQ2 to interact with its primary receptor CD46 and subsequently trigger fusion (14, 33, 34). Although highly speculative, HHV-7 gH/gL associated with either gO or gQ may even provide additional receptor specificity, as was suggested for HHV-6 (Table 1) (35). As such, HHV-7 could employ gH/gL/gO for entry into CD4^+^ cells and gH/gL/gQ for entry into other cell types (Fig. 1).

**TABLE 1:** Comparison of viral ligands and cellular receptors implicated in attachment and entry of three major betaherpesviruses HCMV, HHV-6, and HHV-7^*a*^

	HCMV		HHV-6		HHV-7		
	Viral ligand	Cellular receptor	Viral ligand	Cellular receptor	Viral ligand	Cellular receptor	Reference
Attachment	gB and gM/gN	HSPGs	gQ1/gQ2?	HSPGs?	gB and gQ	HSPGs	(11, 12)
Binding and entry	gH/gL/gO	PDGFR-α	gH/gL/gO	Unknown	gH/gL/gO?	CD4	(11, 17)
	gH/gL/pUL128/pUL130/pUL131A	NRP2	gH/gL/gQ1/gQ2	CD46 (HHV-6A) and CD134 (HHV-6B)	gH/gL/gQ	Unknown	Speculative
	gB	None, EGFR, PDGFRα, integrins	gB	None or unknown	gB	None or unknown	(17)

^*a*^ The former two have been extensively reviewed by Nishimura and Mori (30) and specific references are provided for HHV-7. HSPGs: heparan sulfate proteoglycans; PDGFR-α: platelet-derived growth factor receptor A; NRP2: neuropilin 2; EGFR: epidermal growth factor receptor; ?: research indicates, but does not prove, interaction.

Following herpesvirus de-envelopment, which may occur either at the plasma or endosomal membranes, the nucleocapsid and tegument proteins are released inside the cytoplasm. The nucleocapsid travels towards the nuclear membrane, where it releases viral DNA into the nucleus *via* the nuclear pore complex. In the nucleus, viral transcription is initiated and proceeds *via* a cascade-like manner typical for herpesviruses (36). First, immediate early (alpha) genes are transcribed which encode proteins necessary for the expression of early (beta) genes. Early (beta) gene products regulate viral DNA replication and orchestrate the transcription of the late (gamma) genes encoding multiple viral structural proteins (e.g., capsid, tegument, and envelope proteins) (36, 37). Viral proteins are synthetized in the cytoplasm and capsid proteins reroute to the nucleus for assembly of capsids, prior to encapsidation of the viral DNA. The nucleocapsid then travels *via* the inner and outer nuclear membrane into the cytoplasm (9, 38). Nucleocapsids become decorated with tegument proteins inside the cytoplasm and acquire their envelope by budding into the Golgi apparatus. *In vitro* viral replication in T cells induces a typical cytopathic effect (CPE) characterized by the development of ballooning degeneration and multinucleated giant cells. The giant cells arise from single infected cells undergoing a process of polyploidization and not from the fusion of cells into syncytia as described for other herpesviruses (39). The majority of these multinucleated cells undergo necrotic cell lysis releasing virions in the extracellular space and thus represent a major source of infectious particles (40). Whether virions can also exit their host cell through vesicle-mediated exocytosis, as described for HHV-6, is not known (41). The complete HHV-7 replication cycle takes 3 to 5 days to complete.

## PATHOGENESIS INSIDE THE HOST

**Fig 3:**
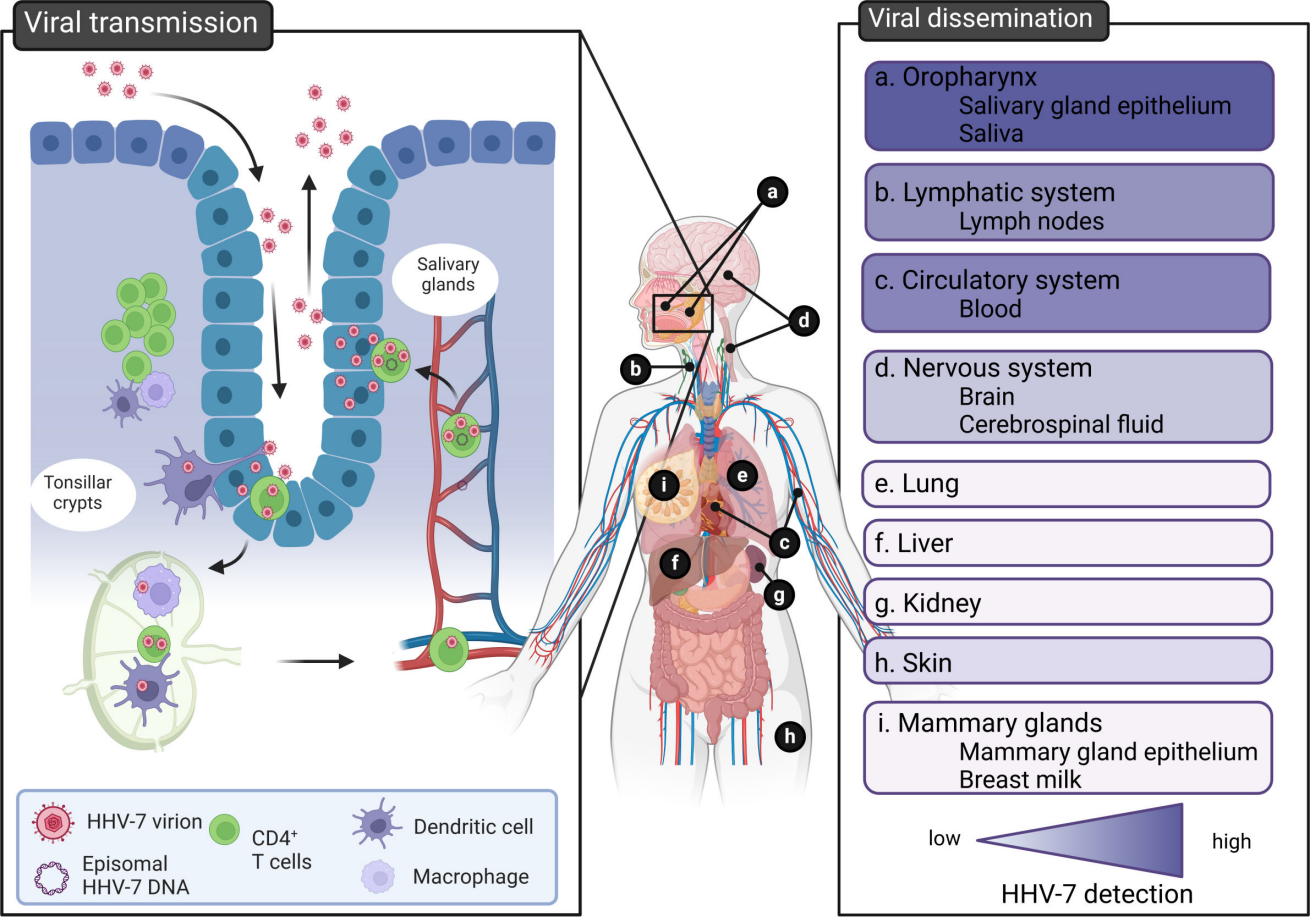
Hypothetical model of HHV-7 pathogenesis inside the human body. HHV-7 transmission (infection and shedding) occurs at the level of the oropharynx and salivary glands (left panel). HHV-7 disseminates to multiple organs inside the host. The gradient color (purple) indicates low to high evidence for HHV-7 detection in this organ, tissue, or bodily fluid (right panel). The figure was created using BioRender.com.

A hypothetical model for HHV-7 pathogenesis inside the human body is depicted in Fig. 3. Primary infection is established upon intake of virus-loaded bodily fluids. The exact portal of entry remains to be fully elucidated but most plausible candidates include the epithelial cells and/or CD4^+^ T lymphocytes and macrophages of the tonsils located in the oral and nasopharyngeal mucosa. As suggested for EBV, viral progeny propagated in epithelial cells may be able to infect immune cells more efficiently and vice versa, fueling primary HHV-7 infection (42). Next, HHV-7-infected immune cells can travel toward draining lymph nodes through the action of HHV-7 U12 and U51. Indeed, these chemokine receptor-like proteins have been shown to interact with chemokine receptor (CCR) 7 agonists, including secondary lymphoid-tissue chemokine (SLC) and EBI1 ligand chemokine (ELC), stimulating homing and trafficking of lymphocytes into and within secondary lymphoid tissues (43). Furthermore, these virally encoded putative chemokine receptors also engage CCR4 agonists including chemokine ligands (CCL) 17 and CCL22 stimulating close interactions between T cells and T cells and macrophages (44). These close interactions could enable cell-associated spread of HHV-7 between neighboring cells, thereby avoiding the release of virus particles into the hostile extracellular environment.

Migration of infected peripheral blood mononuclear cells (PBMCs) into the bloodstream can initiate the viremic phase. Whether these cells actively shed free virus particles in plasma is not known. A previous study showed that plasma-derived viral DNA rather originates from cell lysis and release of viral nucleic acids than from virions (45). In addition, the adaptive immune system would rapidly neutralize free virus particles, suggesting that HHV-7, like other herpesviruses, initiates a cell-associated viremia. Besides, based on the homology between the roseoloviruses, HHV-7 might be able to refrain from viral protein expression at the cell surface and together with other immune-evasive strategies be capable of decoying patrolling immune cells in the blood and lymph system, as has been described for HHV-6.

HHV-7 disseminates to other parts of the body during the viremic phase. Immunohistochemistry studies show that HHV-7 can infect cells that are morphologically and phenotypically distinct from lymphocytes (e.g., dendritic and epithelial-like cells) in multiple tissues including lungs, skin, mammary glands, liver, and kidney (18, 24, 46). Whether productive HHV-7 replication takes place at these secondary sites is not known.

During primary infection and the viremic phase, a majority of infected immune cells will eventually succumb to infection, while other infected immune cells may be “saved” by HHV-7 to function as a life-long latency reservoir (40, 47). These cells still harbor viral DNA but do not produce viral transcripts or viral progeny. In line with this, viral DNA, but not viral transcripts, is frequently recovered from PBMCs of healthy individuals (37). Since resting T cells rarely shed infectious progeny, it is believed that these cells act as a latent reservoir (9, 48). Viral reactivation may occur in case infected T cells become activated, as cognate antigen stimulation results in the onset of HHV-7 lytic replication and release of infectious virus particles (1, 9, 48, 49). Given their susceptibility to HHV-7 and their proven involvement in HCMV and HHV-6 latency, myeloid cells such as dendritic cells and monocytes could potentially also act as a site of latent infection (4, 24, 25, 50). Periodic reactivation allows the virus to transfer the infection to new hosts or fuel latency reservoirs within a host. In comparison with other herpesviruses, HHV-7 reactivation typically occurs during periods of immune suppression (51, 52). Still, HHV-7 reactivation does not always co-occur with immune suppression, as the virus is often detected in healthy individuals (18, 19, 53–64). How the virus exactly transfers infection from immune cells to salivary glands to shed viral progeny into the outer environment is currently unknown. As for EBV, infected leukocytes might reroute to the oro-respiratory tract and produce virions spiked with specific envelope glycoproteins (42, 65). For instance, gH/gL/gQ-pseudotyped virus particles might then be efficiently transferred to (salivary gland) epithelial cells, which could amplify the infection and shed a high viral load in salivary secretions to infect new hosts. Given the homology of U12 and U51 to HCMV U28, a CX3CR1 mimicker that binds fractalkine, fractalkine expression on salivary gland epithelial cells may additionally enhance virion-cell binding and thus the transfer of infection (66). However, Latchney et al. (67) could not identify a correlation between HHV-7 infection and fractalkine expression in human salivary glands, suggesting that fractalkine is not a prerequisite for HHV-7 infection. The majority of cell types residing in the salivary gland epithelium are susceptible to HHV-7, including ductal, cuboidal, and columnar epithelial cells as well as mucous and acinar cells (18, 19, 58, 68).

Multiple researchers suggest that besides the typical periods of herpesvirus latency and reactivation, HHV-7 may adopt a state of persistent infection. The high frequency at which HHV-7 is detected in saliva and salivary gland cells would indicate that persistent HHV-7 infection is established in the mouth (18, 19, 53–64). Still, there is no hard evidence for this hypothesis, as it may also be that reactivation events leading to transfer infection at the level of the salivary glands happen more frequently for HHV-7 compared to other herpesviruses.

## IMMUNE EVASION MECHANISMS

Over years of co-evolution with their respective host, herpesviruses have mastered various strategies to persist in an immunocompetent host population. The large herpesviral genome (145 kilobase pairs for HHV-7) consists of more than a hundred different genes providing an arsenal of viral proteins and noncoding RNAs to counteract the host immune system (69).

One of the most successful immune evasion mechanisms and hallmark of all herpesviruses is the establishment of a lifelong latency in their host following primary infection. Except for transcription of latency-associated genes, latent virus shuts down the transcription of its genome, allowing the virus to stay hidden from the host’s immune surveillance. Upon primary infection, HHV-7 genomes are maintained as episomes in the nucleus of latently infected cells such as resting T cells (20, 37, 48). As described for other betaherpesviruses, HHV-7 may also be able to establish latency in bone marrow-derived hematopoietic progenitor cells (70, 71). The latent stage is sporadically interrupted by periods of lytic replication in a subset of latently infected cells, during which infectious progeny is produced. In turn, this may be transmitted to new hosts or used to restock sites of latent and/or persistent infection. This so-called viral reactivation arises from changing host factors promoting cell differentiation or activation. For instance, T-cell activation and inhibition of apoptosis facilitate the onset of HHV-7 lytic replication (48, 49). The activation state of T cells likely primes HHV-7 genomes for transcription either by stimulation of cellular transcription factors and/or inhibiting histone deacetylases (HDAC) which unwrap chromatin.

Another common herpesvirus strategy HHV-7 utilizes is the downregulation of class I major histocompatibility complex (MHC I) surface expression to avoid cytotoxic T lymphocyte (CTL)-mediated killing of infected cells. To do so, the viral protein U21 associates with class I MHC molecules and a putative Golgi membrane protein or adaptor protein resulting in the sorting of these complexes to lysosomes, where they are degraded (72–78). Cells with reduced MHC I expression at the plasma membrane are normally recognized and cleared by host NK cells. However, HHV-7 circumvents NK-induced cell lysis by simultaneously rerouting NK-activating ligand UL-16 binding protein 1 (ULBP1) to the lysosomal compartment through the action of the same immune-evasion protein U21. In addition, U21 downregulates surface expression of the NK-activating ligands MHC class I polypeptide-related sequences A and B (MICA and B), resulting in the escape from NK-mediated cytotoxicity (79). Finally, U21 downregulates MHC class II proteins, additionally aiding HHV-7 in escaping helper immune cells (77). Notably, the host responds to HHV-7 infection by upregulating IL-15 production, which then results in an enhancement of NK cell activity (80). This is a neat example of the evolutionary arms race between host and virus, where each must counteract the other.

The onset of an adequate immune response may additionally be hampered by the function of HHV-7 U12 and U51 gene products. These viral proteins act as chemokine receptors that may divert chemokines from their natural ligands subverting a local immune response (43, 81, 82). Furthermore, viral replication induces apoptosis in bystander cells through the release of danger signals. For instance, HHV-7-infected cells upregulate the expression of TNF-related apoptosis-inducing ligand (TRAIL) inducing a cytopathic effect on adjacent bystander cells *via* activation of the TRAIL signaling pathway (47). Conversely, HHV-7-infected cells show a marked decrease in surface TRAIL-receptor 1 (TRAIL-R1) expression, thereby avoiding TRAIL-mediated cytotoxicity (47). This favors the survival of infected T cells while neighboring immune cells that may sense the virus are killed, enabling the virus to persist in its host. Even though these HHV-7-infected CD4 T cells are rescued from apoptosis, virus-induced changes perturb the proper immune functions of CD4 cells. For instance, HHV-7 replication in CD4^+^ T cells is accompanied by a downregulation of CD4, CD3, and CXCR4 (27, 83–85). As such, as for HIV, the viral tropism for CD4 T cells itself may act as an immune-evasive strategy by reducing the repertoire of helper T cells *via* lytic replication and other immunomodulatory effects eventually causing immunodeficiency (40, 80).

Finally, direct cell-to-cell spread is another major strategy for HHV-7 to bypass the hostile extracellular environment, which contains phagocytes, antibodies, and complement. Indeed, the virus is best spread *via* cell-cell contact which may be facilitated by U54, as described for HHV-6 (10, 86).

## EPIDEMIOLOGY

HHV-7 specifically infects humans and is common throughout the globe. Specific IgG antibodies against HHV-7 can be found in over 90% of the adult human population (3). As for other herpesviruses, primary HHV-7 infection occurs most commonly in early childhood and lifelong persistence of the virus *via* a combination of latency and ongoing active replication in salivary glands enables the maintenance of a robust immune response for the life of the host (87). Young children become newly seropositive during the decline in maternal antibodies, with approximately 18%–43% of children becoming seropositive within the first year of life. By the second year, this proportion increases to 53%–67%, and by the third year, a substantial majority of children, approximately 93%, have acquired specific antibodies to HHV-7 (3, 88–91). Prevalence rates based on antibody detection are almost universal throughout the world (92, 93). One study reported that seasonal (autumn) and ethnicity factors (Black race) were associated with a higher prevalence of anti-HHV-7 antibody detection in children (94). However, antibody prevalence does not necessarily correlate with active HHV-7 infection and other characteristics associated with socioeconomic status may also have confounded these results.

HHV-7 infection mainly spreads *via* infectious bodily fluids such as saliva and respiratory secretions. Interestingly, an estimated 55% to 90% of people shed infectious HHV-7 intermittently in saliva (18, 19, 53–57, 59–64). This might imply that HHV-7 rather establishes a persistent active infection instead of the typical herpesvirus latency state or that the virus repeatedly reactivates from latency in certain anatomical sites like salivary glands and tonsils (18, 19). Children can acquire the virus from their parents, siblings, or other children (95). Although it has not been proven, mother-to-child transmission may occur during birth or through breast milk. HHV-7 DNA has been detected in breast milk samples and viral proteins have been found in mammary glands (18, 96). However, antibodies to HHV-7 in breast milk may also protect against infection since breastfeeding has been associated with a lower risk of early acquisition of HHV-7 infection (94). Furthermore, HHV-7 DNA has been detected in 3%–10% of cervical swabs obtained from women in their third trimester of pregnancy, but from none of the swabs of non-pregnant control women, suggesting that pregnancy may be associated with reactivation of HHV-7 (97–99). Still, it is unclear whether perinatal transmission can occur through contact with infected maternal secretions, and neonatal infections with HHV-7 have not been reported to date (100). Urine and stool only sporadically contain traces of HHV-7 DNA and are thus unlikely to be a source of transmission (53–55, 101, 102). Finally, HHV-7’s T-lymphotropic character and occasional presence in plasma suggest the possibility of viral transmission during blood transfusions or organ transplantations, but well-documented case reports or series are missing (103–105).

## CLINICAL MANIFESTATIONS

It is often difficult to identify direct causality between herpesviruses and clinical manifestations due to the ubiquitous nature of herpesviruses and their capacity to induce a lifelong infection where only certain individuals experience problems either through direct cytopathology or by triggering a pathological immune response (87). Therefore, we have used a set of criteria based on the revised postulates of Koch that were suggested by Komaroff et al. (106), to evaluate associations between HHV-7 and different clinical manifestations (Tables 2 and 3).

**TABLE 2:** Criteria helpful in evaluating the causal role of HHV-7 in dermatological diseases, based on the revised postulates of Koch suggested by Komaroff et al. (106)^*a*^

	Roseola infantum	Pityriasis rosea	Atypical exanthem	PPGSS	DIHS/DRESS	TEN	Lichen planus
HHV-7 nucleic acid is present in diseased tissue/individuals.	Blood (89, 102, 107, 108)	Blood and skin (109–112) Negative evidence of blood and skin (113–115)	Blood (116) Negative evidence skin (116)	Blood (116–118) Negative evidence skin (116)	Blood and skin (119–123)	Throat swab (124)	Skin (24, 46, 125–127)
The amount of HHV-7 nucleic acid in diseased tissue and/or antibody levels correlates with the severity of the disease.	Nucleic acid and antibody levels (89, 107, 108, 128, 129)	Nucleic acid and antibody levels (110, 111, 117, 130) Negative evidence of nucleic acid and antibody levels (113–115)	No evidence	Antibody levels (118, 131)	No evidence	Negative evidence of antibody levels (132)	Nucleic acid levels (24, 46, 125, 127)
HHV-7 mRNA, antigens, or infectious virions are present in diseased tissue.	Antigens and infectious virions (89, 102, 107, 108, 129)	mRNA and antigens (110, 111)	No evidence	No evidence	No evidence	No evidence	Antigens (24, 46)
Exposure to and then the presence of the viruses and their gene products in affected tissue precede the development of the disease or seroconversion is detected (temporal relationship).	Seroconversion (89, 107, 108, 128, 129)	Negative evidence seroconversion (112)	No evidence	No evidence	Seroconversion (119–121, 123)	T-cell immunity (124) Seroconversion (133)	Nucleic acids and antigens disappear upon remission (46)
Infectious agents other than HHV-7 are not generally detected in diseased tissue in a substantial number of cases.	Positive evidence (107, 129) Negative evidence (HHV-6) (89, 102, 108, 128)	Positive evidence (111) Negative evidence (HHV-6) (110, 114)	Negative evidence (other viruses, bacteria, and parasites) (134)	Positive evidence (116, 117) Negative evidence (Parvovirus B19) (117, 118)	Positive evidence (119, 121) Negative evidence (other herpesviruses)(119, 120, 123, 133)	Negative evidence (other herpesviruses, coxsackievirus A6, and bacterial infections) (124, 133)	Positive evidence (24) Negative evidence (126)
HHV-7 affects cellular function in diseased tissue in a manner able to cause or augment the disease pathology (*in vitro* or *in vivo* studies).	Lymphocyte CPE (89, 102)	Lymphocyte CPE (109)	No evidence	No evidence	No evidence	No evidence	No evidence
Specific antiviral therapy reduces viral load in diseased tissue or blood and is followed by clinical improvement.	No evidence	Positive evidence (135–137)	No evidence	No evidence	No evidence	No evidence	No evidence

^*a*^ All evidence cited is positive in support of the assertion unless specifically identified as negative evidence. PPGSS: papular purpuric gloves and socks syndrome; DIHS: drug-induced hypersensitivity syndrome; DRESS: drug reaction with eosinophilia and systemic symptoms; TEN: toxic epidermal necrolysis; CPE: cytopathogenic effect.

**TABLE 3:** Criteria helpful in evaluating the causal role of HHV-7 in neurological diseases, based on the revised postulates of Koch suggested by Komaroff et al. (106)^*a*^

	Febrile seizures/epilepsia	Encephalitis	Meningitis	Myelitis	Neuritis	Hippocampal sclerosis	Meningo-/myelo-radiculopathy
HHV-7 nucleic acid is present in diseased tissue/individuals.	Blood (89, 128, 138–140) CSF (6, 140–144)	Blood (140, 145, 146) CSF (6, 140–143, 146–158) Brain tissue (22, 153, 159)	Blood (140) CSF (140, 141, 147, 148, 156, 160–162)	CSF (141, 147, 148, 161, 163, 164)	CSF (141, 144, 160, 165)	Hippocampus (166)	CSF (147, 151, 165, 167, 168)
The amount of HHV-7 nucleic acid in diseased tissue and/or antibody levels correlates with the severity of the disease.	Nucleic acid levels (145)	Nucleid acid and antibody levels (141, 145, 148, 22, 159, 169)^129^, ^146^	Nucleid acid levels (141)	No evidence	Nucleid acid levels (141)	Nucleic acid levels (166)	No evidence
HHV-7 mRNA, antigens, or infectious virions are present in diseased tissue.	mRNA and infecitous virions (138, 139)	Antigens (22)	mRNA (161)	mRNA (161)	No evidence	Antigens (166)	No evidence
Exposure to and then the presence of the viruses and their gene products in affected tissue precede the development of the disease or seroconversion is detected (temporal relationship).	Seroconversion (128, 145)	Seroconversion (146, 147, 149–151, 170)	Seroconversion (160)	No evidence	Seroconversion(160, 165)	No evidence	Seroconversion (147, 151, 165)
Infectious agents other than HHV-7 are not generally detected in diseased tissue in asubstantial number of cases.	Positive evidence (129, 139, 140, 144) Negative evidence (HHV-6) (128, 138, 145)	Positive evidence (22, 140, 144, 146, 151, 152, 155, 158) Negative evidence (145, 148, 154, 156, 157, 159)	Positive evidence (140, 160, 162) Negative evidence (156)	Positive evidence (163)	Positive evidence (144, 160) Negative evidence (165)	No evidence	Positive evidence (151, 168) Negative evidence (165)
HHV-7 affects cellular function in diseased tissue in a manner able to cause or augment the disease pathology (*in vitro* or *in vivo* studies).	Lymphocyte CPE (89, 102)	No evidence	No evidence	No evidence	No evidence	No evidence	No evidence
Specific antiviral therapy reduces viral load in diseased tissue or blood and is followed by clinical improvement.	Positive evidence (6, 143, 144)	Positive evidence (6, 143, 144, 148, 152, 154)	Positive evidence (148, 161)	Positive evidence (163)	Positive evidence (144)	No evidence	Negative evidence (167)

^*a*^ All evidence cited is positive in support of the assertion unless specifically identified as negative evidence. CSF: cerebrospinal fluid; CPE: cytopathogenic effect.

### Dermatological diseases

HHV-7 has been linked to a number of dermatological diseases, although its role in the pathophysiology of these illnesses is not fully understood.

HHV-7, like HHV-6, has a proven association with roseola infantum, also known as exanthem subitem or sixth disease, although HHV-7 is less frequently linked to the disease compared to HHV-6 (102, 107, 108, 128, 129). Exanthem subitum is a common childhood illness that mostly develops before the age of 3 and is non-discriminatory in gender and location. Around 50% of HHV-7 infections in children induce exanthem subitem and symptoms vary from absence to a fever and/or a rash that lasts one to several days (128, 129). The rash is characterized by non-pruritic papules and macules and typically starts on the trunk and can spread to the neck, extremities, and face. Other symptoms include anorexia, leukopenia, mild diarrhea, palpebral edema, mild inflammation of the pharynx, and mild occipital and cervical lymphadenopathy. Serious complications are rare but may include febrile seizures and/or status epilepticus (89, 138). Febrile seizures occur in 2%–5% of children younger than the age of 5 and around 7% of these cases can be linked to HHV-7 viremia (108, 138). For HHV-6, these febrile seizures have been linked to a dysfunctional blood-brain barrier caused by virus-induced rises in serum matrix metalloproteinases (171). Whether this also occurs during HHV-7 infection has not been studied. Most cases of roseola infantum improve on their own. Virus replication in the naso- and oropharynx and/or draining lymph nodes along with the viremic phase account for most symptoms. Histopathological examination of viral exanthem usually shows normal epidermis with sparse perivascular infiltration of lymphocytes and/or vasculitis (172).

As shown in Table 2, a more debated association of both HHV-7 and HHV-6 is pityriasis rosea, a common skin rash with a prevalence of 1.3% that typically occurs in young adults, usually lasts less than 3 months and disappears without treatment (109, 110, 113–115, 130, 173). The condition often starts with a single, slightly raised, scaly patch called the “herald patch” on the torso, followed by the appearance of smaller similar patches on the torso and extremities. HHV-7 antigens and DNA have been detected in up to 83% of skin lesions of pityriasis rosea and to a lesser extent in other dermatites (109–111, 117, 174, 175). Furthermore, higher viral loads in PBMCs and/or plasma are observed in cases of pityriasis rosea compared to controls. However, viral DNA and antigens can also be retrieved from non-lesional skin or control subjects, and it is not always easy to distinguish latent from active viral replication (110, 113, 174). Therefore, the exact role of HHV-7 in the pathogenesis of pityriasis rosea is still up for debate. An association seems likely, but the etiologic mechanism remains unknown.

The presence of HHV-7 has also been linked to several other dermatitis including atypical exanthems (116), papular purpuric gloves and socks syndrome (PPGSS) (116–118, 131), drug-induced hypersensitivity syndrome (DIHS) or drug reaction with eosinophilia and systemic symptoms (DRESS) (119–123, 133), immune-mediated toxic epidermal necrolysis (124, 133), and lichen planus (24, 46, 125–127). The extent to which HHV-7 infection directly contributes to these syndromes acts as an exogenous antigen in immune reactions, or if HHV-7 reactivation is simply a side reaction to the disease remains largely unknown (Table 2).

### Neurological disorders

As described above, seizures are not an uncommon complication of HHV-7 infection and are often associated with viral-induced high fever (i.e., febrile seizures) (89, 128, 139, 141, 142). Congruent with febrile seizures, HHV-7 viremia has also been associated with febrile status epilepticus (138). One study also linked the presence of HHV-7 DNA and antigens in the brain to inflammatory-mediated hippocampal sclerosis and drug-resistant epilepsy (166). Other neurological disorders such as encephalitis, meningitis, myelitis, cerebellitis, neuritis, and meningo- or myeloradiculopathy (e.g., Guillian Barré syndrome) have also been observed during ongoing HHV-7 infection (6, 128, 129, 139–141, 143–158, 160–165, 167, 168, 170). In most cases, CNS manifestations ranging from nausea, sensitivity to light, and a stiff neck to ataxia and paralysis were accompanied by the detection of HHV-7 nucleic acids in cerebrospinal fluid (CSF) and/or synthesis of intrathecal anti-HHV-7 antibodies (6, 129, 140, 141, 143–158, 160–164, 167, 168). Of note, HHV-7-specific antibodies or DNA were usually not accompanied by the presence of other viral DNA or antibodies, ruling out potential leakage through the blood-brain barrier (BBB) and indicating that HHV-7 can invade the nervous system. In addition, multiple studies have detected HHV-7 DNA and antigens in the brains of persons with and without neurological pathologies (22, 153, 159, 166, 169). More precisely, HHV-7 DNA has been retrieved from the meninges (dura mater and pia mater) (159), frontal lobe (22, 159, 169), temporal lobe (22, 159, 169), occipital lobe (169), parietal lobe (169), hippocampus (159, 166), olfactory tract (159), optic tract (159), cerebellum (169), and brain stem (153). Viral proteins have been reported in astrocytes, oligodendrocytes, as well as neurons (22, 166). How exactly HHV-7 reaches the brain parenchyma is unknown, but this presumably occurs either *via* retro- and anterograde viral transport through peripheral nerves (e.g., olfactory or optic tract) or *via* the vascular system where the virus passes through the BBB either cell-free or cell-associated. Upon reaching the nervous system, local viral replication with accompanying damage and/or vasculitis accompanied by a focal impairment of blood flow can cause neurological damage resulting in neurological disease. Alternatively, as an exogenous antigen, HHV-7 may also be a pathological factor in the development of immune-related neurological damage.

The above-described case studies suggest, but do not prove, a neurotropic and neuropathogenic potential of HHV-7 (Table 3). Still, unlike HHV-6, HHV-7 is not a common cause of encephalitis and *in vitro* replication in neuronal cell lines has not been reported (176). The development of neurological disease is likely multifactorial depending not only on the viral strain but also on host factors such as age and immune status. As described for other herpesviruses, primary HHV-7 infections delayed into adolescence might cause more severe neurological diseases than those occurring in early childhood (140, 143, 144, 147, 149, 155, 168). This is because the aggressive inflammatory response produced by a more mature immune system can paradoxically lead to more tissue damage. Conversely, the inability of the immune system to locally contain HHV-7 infection in immunocompromised individuals [e.g., corticosteroids, chemotherapy, transplantation, human immunodeficiency virus (HIV) infection] also predisposes patients to more severe neurological diseases (153, 154, 160, 163, 164).

### Other clinical associations

HHV-7 infection has been linked to various clinical syndromes not only in individuals undergoing transplantations but also in non-transplant settings.

Transplantations are preceded by aggressive conditioning regimens that deplete existing bone marrow and immune cells. Suppression of the recipient’s immune system is necessary to maximize the chances of engraftment and long-term function of the transplanted organ or cells. As stated above, immune suppression may evoke reactivation events of endogenous herpesviruses or predispose patients to acquiring (re)infections from infected individuals or even donor transplants. HHV-7 reactivation or (re)infection has been linked to various complications in transplant recipients with or without other concomitant infections, including CNS disease (see above), hepatitis, bronchiolitis, pneumonia, transplant rejection, and CMV disease (177–183). These case studies have associated HHV-7 with transplant complications based on the detection of HHV-7 DNA in either the blood or CSF of the patients but do not describe the underlying mechanisms. Furthermore, the exact incidence of specific HHV-7-induced transplant complications remains uncertain.

HHV-7 infection has also been implicated in diverse clinical syndromes beyond the context of transplantations and in immunocompetent hosts, including mononucleosis-like illnesses (184–187), acute respiratory distress syndrome and interstitial pneumonia (188, 189), hepatitis (190), myocarditis (191, 192), fibromyalgia (193), connective tissue disease (194), and periodontitis (195). In these case studies, HHV-7 diagnosis was based on seroconversion and/or detection of HHV-7 DNA in several anatomical compartments (blood, lungs, BAL, liver biopsies, etc.). Still, whether the viral DNA derives from circulating blood-derived PBMCs or tissue-resident cells is unclear. Currently, the causative role of HHV-7, either alone or in conjunction with other viruses/factors, in causing these syndromes, remains solely speculative, as proving causation remains complicated, partially due to the regular detection of HHV-7 in healthy people.

## CONCLUSIONS, KNOWLEDGE GAPS, AND RECOMMENDATIONS FOR FUTURE RESEARCH

Despite its initial identification in 1990, HHV-7 remains an understudied herpesvirus ominously present in the human population. HHV-7, like other herpesviruses, typically presents minimal or no issues when acquired naturally during early childhood and remains in a state of equilibrium with its host. However, a slight disruption in this equilibrium, such as delayed infections occurring during adolescence or immune suppression, can shift the balance toward a more pronounced and severe clinical outcome. Still, little is known about the etiological nature of most of these manifestations. To better understand the critical interplay between virus and host, we need to gain more insights in viral pathogenesis. More precisely, studies should investigate how and where HHV-7 replicates and hides inside its host and how the host immune system responds to incoming viruses. This information could reveal triggers of specific clinical syndromes of severe HHV-7-induced manifestations, leading to the identification of new cures, treatments, and/or prevention strategies, ultimately benefitting patients.

One of the major limitations in HHV-7 research is the species-specific nature of HHV-7 and thus the lack of suitable *in vivo* models to study the viral pathogenesis. Unfortunately, well-controlled inoculation experiments in naïve hosts cannot be tested ethically in patients and, therefore, we must rely only on case series and *in vitro* models. Still, case studies lack a well-controlled experimental setup where the early phase of infection has usually already passed upon clinical presentation, and invasive sampling to study viral dissemination simply cannot be done. Furthermore, the complex interplay between HHV-7-infected and neighboring cells in a 3D environment, as well as the inflammatory processes triggered by HHV-7 cannot be accurately recapitulated *in vitro*. Still, there are solutions and alternatives to explore HHV-7 pathogenesis in animal models. First, as for HIV, a humanized mouse model in which human immune cells are engrafted could potentially be used to study HHV-7 infection, as described for HHV-6 (196). Notably, viral transfer between different anatomical compartments cannot be replicated in the latter model, since non-immune cells (e.g., neurons and epithelial cells) remain mouse-derived and might not support viral replication. Inoculating mice with a mouse-specific roseolovirus closely related to HHV-6 and HHV-7 (e.g., murine roseolovirus or MRV) might be an interesting substitute to broaden insights into HHV-7 immunopathogenesis (197). Similarly, murine CMV is used to mimic HCMV pathogenesis in mice (198). Alternatively, pigtailed macaque roseolovirus or *Macaca nemestrina* herpesvirus 7 (MneHV7) is another roseolovirus that even more closely resembles HHV-7 than MRV and could be used to infect non-human primates (199). Besides *in vivo* models, *ex vivo* models where a 3D architecture between different cell types is reconstructed, (e.g., explant, organoid, transwell, and trichamber models) could also partly mimic the interplay between epithelial cells and immune cells or even construct segmented environments between different cell types to study viral transfer infection (200, 201).

Finally, our review also identified many knowledge gaps in the HHV-7 life cycle, especially the entry step. With the rise of versatile gene-editing tools such as CRISPR-Cas9, new viral mutants, and cellular gene knockouts could more easily be generated to further unravel these steps (200). Identifying additional receptors might, for instance, provide new targets for cure interventions in severe clinical manifestations related to HHV-7 infection (e.g., neurological disorders and transplant complications).

Together, HHV-7 has been associated with a variety of clinical syndromes suggesting it has a broader impact on human health than previously thought. However, new *ex vivo* and *in vivo* experiments are urgently needed to broaden our insights into the viral pathogenesis and find new intervention strategies.
